# Domain Generalization for Language-Independent Automatic Speech Recognition

**DOI:** 10.3389/frai.2022.806274

**Published:** 2022-05-12

**Authors:** Heting Gao, Junrui Ni, Yang Zhang, Kaizhi Qian, Shiyu Chang, Mark Hasegawa-Johnson

**Affiliations:** ^1^Department of Electrical and Computer Engineering (ECE), Beckman Institute, University of Illinois, Urbana, IL, United States; ^2^MIT-IBM Watson AI Lab, Cambridge, MA, United States; ^3^Department of Computer Science, University of California, Santa Barbara, Santa Barbara, CA, United States

**Keywords:** automatic speech recognition, under-resourced languages, invariant risk minimization, distributionally robust optimization, regret minimization, domain generalization

## Abstract

A language-independent automatic speech recognizer (ASR) is one that can be used for phonetic transcription in languages other than the languages in which it was trained. Language-independent ASR is difficult to train, because different languages implement phones differently: even when phonemes in two different languages are written using the same symbols in the international phonetic alphabet, they are differentiated by different distributions of language-dependent redundant articulatory features. This article demonstrates that the goal of language-independence may be approximated in different ways, depending on the size of the training set, the presence vs. absence of familial relationships between the training and test languages, and the method used to implement phone recognition or classification. When the training set contains many languages, and when every language in the test set is related (shares the same language family with) a language in the training set, then language-independent ASR may be trained using an empirical risk minimization strategy (e.g., using connectionist temporal classification without extra regularizers). When the training set is limited to a small number of languages from one language family, however, and the test languages are not from the same language family, then the best performance is achieved by using domain-invariant representation learning strategies. Two different representation learning strategies are tested in this article: invariant risk minimization, and regret minimization. We find that invariant risk minimization is better at the task of phone token classification (given known segment boundary times), while regret minimization is better at the task of phone token recognition.

## 1. Introduction

Speech production is a nonlinear process. Any given articulatory movement—say, a shift of 1cm in the position of the tongue tip—may cause a huge change in the produced acoustic spectrum, or a miniscule change, depending on the articulatory position from which the movement started. Let's use the words “unstable” vs. “stable,” respectively, to denote articulations from which small deviations cause large vs. small acoustic consequences. A learner imitating adult speech tends to have greater success in imitating stable rather than unstable articulations, because stability permits accurate acoustic imitation despite imprecise articulatory imitation. For this reason, phonemes tend to correspond to stable articulations, and unstable articulations tend to mark the boundaries between pairs of phonemes (Stevens, 1972). The number of unstable configurations is larger than the number of phoneme distinctions in any known language, therefore each language chooses a subset to use as phoneme boundaries, e.g., some languages treat the phones /θ/ (as in “thin”) and /s/ (as in “sin”) as distinct phonemes, while in other languages, they are both considered to be acceptable pronunciations of the same phoneme. A language-independent ASR is an automatic speech recognizer trained to recognize all of the articulatory features that may be used to signal phoneme distinctions, in any of the world's languages.

The relationships among phoneme inventories of different languages are complicated, however, by tremendous cross-lingual divergence in the use of redundant features (Stevens et al., 1986). No language uses all of the available articulatory features to define phonemes; hence, every language has some extra articulatory features left over, that can be used to add redundancy to its phoneme code. In modern English, for example, the feature of plosive voicing (/d/ vs. /t/) is often enhanced by the feature of aspiration (/d/ vs. /t^h^/), while the tense-lax vowel distinction (/i/ vs. /ɪ/) is often enhanced by the feature of lengthening (/i:/ vs. /ɪ/). In both of these cases, it is possible to identify one feature as *phonemic* and another as *redundant* because, in each case, the redundant feature can be modified without changing the meaning of the word (/bi:t^h^/ and /bit/ are both “beat”). Redundant features add robustness to speech in much the same way that an error-correcting code adds robustness to digital communication systems: imprecise production or noisy perception are less likely to cause communication errors if every phoneme is redundantly specified. Because redundant features improve the efficiency of speech communication, they are ubiquitous.

Because redundant features are defined separately for every language, however, they cause significant problems for the training of language-independent ASR. A typical ASR training corpus is a set of labeled examples, $D=\left\{\left(x_{1},y_{1}\right),\ldots ,\left(x_{n},y_{n}\right)\right\}$, where *x*_*i*_ ~ *X* is a speech waveform, and *y*_*i*_ ~ *Y* is the corresponding text transcript.^1^ We can safely assume that certain transformations are information-preserving, e.g., a waveform can be converted to or from a spectrogram without loss of information (Nawab et al., 1983), therefore we can consider both to be equivalent representations of the random variable *X*. Similarly, in any well-resourced language, a pronunciation lexicon can be used to convert text transcripts to phoneme transcripts encoded using the international phonetic alphabet (IPA Association, 1999), therefore we can consider text transcripts and IPA phonemic transcripts to be equivalent representations of the random variable *Y*. The key obstacle to language-independent ASR is that phonemic transcripts are not the same as language-independent phonetic transcripts. The English word “beat,” for example, has the same phonemic transcript (*y*_*i*_=[bit]), regardless of whether or not the vowel is lengthened (/bi:t/ vs. /bit/), and regardless of whether the final consonant is aspirated, unreleased, glottalized, or replaced by a glottal stop (/bit^h^/, /bit˺/, /bitˀ/, or /biʔ/). The phonetic sequences /bi:t^h^ət/ and /biʔət/ are different words in Arabic (“home” and “environment,” respectively), but an ASR trained using English data would be unable to distinguish them. Similarly, a plosive voicing detector trained on English fails to correctly recognize Spanish unvoiced plosives, which are not aspirated (Lisker and Abramson, 1964), or Hindi voiced aspirated and unvoiced unaspirated plosives (Patil and Rao, 2016). A vowel classifier trained on English is able to recognize the duration differences of some Japanese vowel pairs, but not others (Nishi et al., 2008). A Mandarin vowel classifier, applied to English vowels, finds American English /u/ to be closer to the Mandarin central unrounded vowel /ɨ/ than to the Mandarin /u/ (Shi et al., 2019).

**Figure 1:**
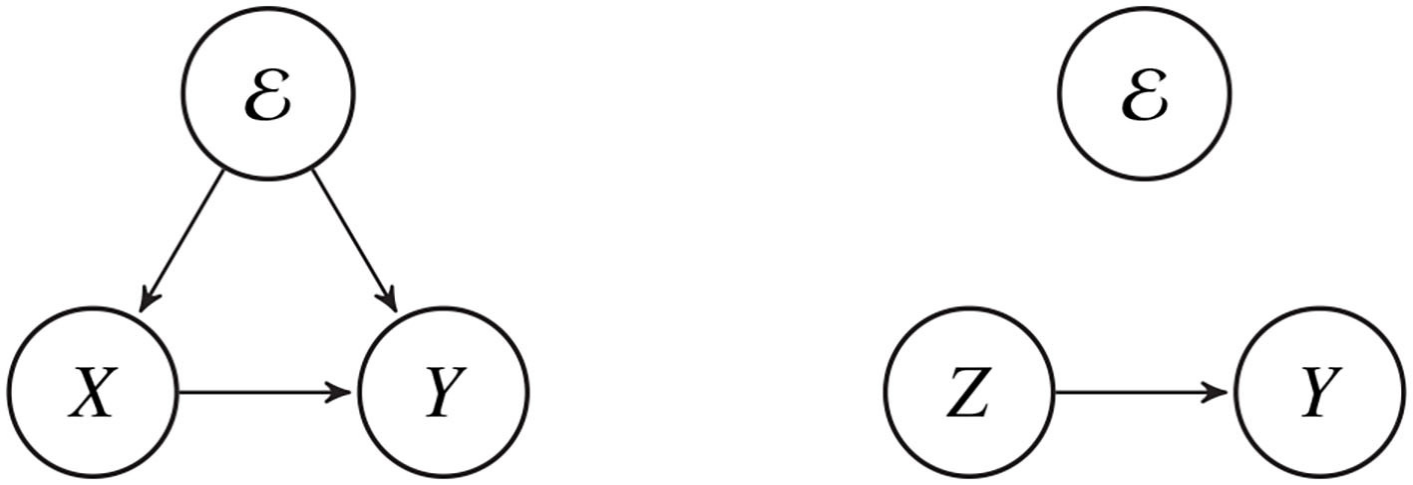
The phonemic transcript, *Y*, captures a limited set of information about the speech signal, *X*. The limits of the transcription process are dependent on the language environment, E. Language-independent ASR finds a feature embedding, *Z* = ϕ(*X*), such that the relationship between *Z* and *Y* is independent of E.

Apparently, what is needed is some type of intermediate representation, capable of compensating for language-dependent differences in the use of redundant features. This article proposes the use of an **invariant embedding**, *z* ~ *Z*, defined to be a high-dimensional signal representation with no information about the language-dependent redundant articulatory features. The invariant embedding allows us to train a language-independent ASR using a large number of language-dependent training corpora. Each language-dependent training corpus contains a number of tuples of the form $D=\left\{\left(x_{1},e_{1},y_{1}\right),\ldots ,\left(x_{n},e_{n},y_{n}\right)\right\}$, where $e_{i}\in E$ specifies the language and dialect being spoken, and the transcriptions are language-dependent phonemic transcripts rather than language-independent phonetic transcripts: *y*_*i*_ = *f*(*x*_*i*_, *e*_*i*_). The invariant embedding is trained to ignore language-dependent redundant features in *X*, and to encode only the features that correspond to *Y* in a language-independent way, so that the mapping *w* : *Z* → *Y* is a language-independent ASR (Figure 1).

For example, suppose that *x*_1_ and *x*_2_ are two different waveforms, each examples of the English word “beat,” meaning that they both have exactly the same label sequence, *y*_1_ = *y*_2_=[bit]. Suppose that fine phonetic transcriptions of these two waveforms would detect some differences, e.g., perhaps *x*_1_ sounds like /bi:t^h^/, while *x*_2_ sounds like /biʔ/. The purpose of the invariant embedding is to eliminate these fine phonetic differences, so that if one were to convert the invariant embedding back into an acoustic signal, the language-independent fine phonetic transcription of that acoustic signal would be exactly the sequence /bit/. In this way, a language-independent speech recognizer, capable of mapping *f* : *X* → *Y*, is decomposed into two subsystems: (1) a feature extraction system computes features *Z* = ϕ(*X*) such that (2) the mapping *w* : *Z* → *Y* is independent of the language environment.

Suppose that an ASR is trained using data from several different training environments $D=\left\{e_{1},\ldots ,e_{K}\right\}\subset E$. Recent survey papers in machine learning and computer vision (Wang et al., 2021; Zhou et al., 2021) usefully distinguish several different ways in which the test environment, $e_{K+1}\in E$, may be related to the training environments. Multi-task or multi-domain learning is the task of optimizing *f*(*X*) so that it performs well for all of the languages in the training set ($e_{K+1}\in D$). Transfer learning and domain adaptation assume that the test language is not in the training set ($e_{K+1}\notin D$), but that a small quantity of labeled data exist in the test language, and that these data can be used to adapt *f*(*X*). Zero-shot learning and domain generalization assume that $e_{K+1}\notin D$, and that furthermore, no data exist for the test environment. Obviously, zero-shot learning and domain generalization are only well-defined problems if we make some *a priori* assumptions about the test environment. For example, we may assume that the training and test environments, {*e*_1_, …, *e*_*K*_, *e*_*K*+1_}, are drawn i.i.d. from the unknown set of all possible environments (E). Multi-task learning, transfer learning and zero-shot learning usually focus on differences between the labels used in training and test environments (*P*_1_(*Y*|*X*), …, *P*_*K*_(*Y*|*X*) ≠ *P*_*K*+1_(*Y*|*X*)), while multi-domain learning, domain adaptation and domain generalization also consider a possible shift between the two feature distributions (*P*_1_(*X*), …, *P*_*K*_(*X*) ≠ *P*_*K*+1_(*X*)).

A recent survey paper (Wang et al., 2021) categorizes approaches to domain generalization into three broad categories, composed of nine subcategories: data manipulation (including augmentation and generation), learning strategies (including ensemble learning, meta-learning, gradient operations, distributionally robust optimization, and self-supervised learning), and representation learning (including feature disentanglement and domain-invariant representation learning strategies). Of these nine, data augmentation (Feng et al., 2021), self-supervised learning (Conneau et al., 2020), and domain-invariant representation learning (Swietojanski et al., 2012) have been used to train ASR for cross-lingual domain generalization, while data generation (Novitasari et al., 2020), ensemble learning (Sahraeian and Compernolle, 2018), meta-learning (Hsu et al., 2020), and gradient operations (Tong et al., 2018) have been used for cross-lingual domain adaptation. Distributionally robust optimization (DRO) has been used to learn ASR that generalizes successfully across gender, age, education, race, or regional dialect of the speaker (Gao et al., 2022), while feature disentanglement has been used to generalize ASR across acoustic domains (Hsu and Glass, 2018), but to our knowledge, neither DRO nor feature disentanglement has ever yet been used for cross-lingual ASR.

This article describes a sequence of experiments intended to test the following hypotheses. Not all of these hypotheses were experimentally verified; the experimental truth or falsehood of each hypothesis is noted briefly here, and is supported by the evidence presented in the remainder of the article.

- **H1:** Domain-invariant machine learning methods such as invariant risk minimization (IRM, Arjovsky et al., 2019), and/or regret minimization (RGM, Jin et al., 2020) can be used to optimize an end-to-end (E2E) neural network ASR so that it more effectively generalizes from fifteen training languages to five novel test languages, as compared to a baseline ASR trained using a standard training criterion called empirical risk minimization (ERM). **Experimental result:** this hypothesis is demonstrated to be false by the experiments presented here.
- **H2:** IRM and/or RGM, as compared to ERM, can be applied to optimize an E2E ASR so that it more effectively generalizes from training languages in one language family to test languages in a different language family. **Experimental result:** true.
- **H3:** The optimal training regimen for phone token classification (given known phone token boundary times) is different from the optimal training regimen for phone token recognition (with unknown boundary times). **Experimental result:** true. Experiments described in this article find that either empirical risk minimization (ERM) or regret minimization (RGM) are optimal for recognition, while invariant risk minimization (IRM) is optimal for classification.

Section 2 discusses background literature, including the ERM, IRM and RGM training strategies. Section 3 describes the adaptation of these training strategies to the task of language-independent ASR. Section 4 describes experimental methods, Section 5 presents results, Section 6 discusses our findings, and Section 7 concludes.

## 2. Background

This section reviews several recent lines of inquiry into the problem of invariant representations. We will normalize all discussion into a common notational scheme. We use a random variable *X* to represent speech data. Each sample *x* ~ *X* is a sequence of raw acoustic feature vectors. We use a random variable *Y* to represent the phoneme transcriptions of speech data. Each *y* ~ *Y* is a sequence of IPA symbols. We use E to denote the set of environments in the training data. In this article, each language is viewed as an environment e∈E, which determines the distributions of acoustic features and phonemes. We use *f* : *X* → *Y* to denote the speech recognition model that takes acoustic features as input and transcribes them into phoneme transcriptions. The model *f* can be viewed as a composition of two parts *f*: = *w* ∘ ϕ, where ϕ:*X* → *Z* : is the feature extractor that maps the raw acoustic feature *X* into a latent representation space *Z*, and *w* : *Z* → *Y*: is a classifier that maps the latent representation *Z* to IPA symbol sequence *Y*. We use R to denote the empirical risk over the entire dataset and use $R^{e}$ to denote the empirical risk over the subset of data from environment *e*.

The remainder of this section is organized according to the three broad categories of domain generalization described in a recent review article (Wang et al., 2021): data manipulation, learning methods, and representation learning. The methods of invariant representation learning, which are the focus of this article, are described in Section 2.3; baseline methods are described in Sections 2.1 and 2.2.

### 2.1. Data Manipulation for Domain Generalization

Risk is defined to be the expected value of loss (Vapnik, 1998). The loss we incur when the utterance (*x*_*i*_, *y*_*i*_) is (mis)recognized as *f*(*x*_*i*_) is measurable using a loss function $L\left(f\left(x_{i}\right),y_{i}\right)$. Risk is therefore computed as the average over all (*x, y*) ~ (*X, Y*):


(1) $$\begin{array}{l} R\left(f\left(X\right),Y\right)=𝔼\left[L\left(f\left(X\right),Y\right)\right]. \end{array}$$

Empirical risk minimization (ERM) minimizes the average loss on the training set, with the goal of achieving high accuracy on an independent and identically distributed test set. ERM is formulated as follows:


(2) $$\begin{array}{l} f_{ERM}=\underset{f}{\mathrm{argmin}}R\left(f\left(X\right),Y\right) \end{array}$$

In the limit of infinite training data, ERM provably minimizes the expected risk on the test corpus, provided that the test corpus and training corpus are drawn from the same distribution (Vapnik and Chervonenkis, 1971). In many practical settings, however, the test corpus and training corpus are not drawn from the same distribution. For example, available ASR training corpora are heavily biased in favor of a few well-resourced languages. During testing, the mixture of languages may be quite different: some languages that were badly under-represented during training may be somewhat more frequent during testing. We can characterize the problems with ERM by separately measuring the risk for each language, e∈E, as


$$\begin{array}{l} R^{e}\left(f\left(X\right),Y\right)={𝔼}_{e}\left[L\left(f\left(X\right),Y\right)\right], \end{array}$$

where 𝔼_*e*_[·] denotes expectation over data drawn from environment *e*.

The error rate of a classifier trained to perform in one environment, then adapted to another environment, has been extensively studied. For example, it is known that knowledge transfer from a source environment to a target environment is beneficial if knowledge of the source environment reduces the Vapnik-Chervonenkis (VC) dimension of the hypothesis space in which the target environment is known to exist (Vapnik, 1998). The VC dimension of a deep network is *O*{*WL*log(*W*)}, where *W* is the number of weights, and *L* is the number of layers (Harvey et al., 2017), therefore the benefit of transfer learning can be measured by the number of layers in the deep network that are transferred from the source environment to the target environment without retraining. One of the reasons for the deep learning revolution was early experimental evidence supporting the claim that, for many common transfer learning tasks, most of the layers can be transferred without retraining (Bengio, 2012); for many tasks it has been reported that pre-training using an unsupervised learning criterion (Salakhutdinov and Murray, 2008) or using a supervised criterion such as ERM (Yosinski et al., 2014) may be remarkably effective.

Several recent papers have demonstrated that domain generalization can be achieved by training a machine learning algorithm using ERM on a sufficiently diverse set of training environments. Many classic papers on domain generalization assume that the set of environments, E, can be neither parameterized nor bounded, but several recent empirical papers have pushed back against that assumption by collecting a very large number of environments, and by training a deep network that shows remarkable empirical ability to generalize to unseen test environments. In one paper (Gulrajani and Lopez-Paz, 2020), a number of domain generalization tasks previously described in the computer vision literature (digit, object and scene recognition tasks with train-test mismatch in terms of object category, color, rotation, environment, obstruction, and rendering style) were attacked using deeper networks, and using modern data augmentation strategies. Results suggest that the deeper network, trained using ERM with random data augmentation during training, performs as well as a comparably deep network trained using any of a large number of learning algorithms or data representations designed to explicitly encourage domain invariance. Similarly, a large experimental study of domain generalization for ASR (Narayanan et al., 2018) collected data from six training domains (Voicesearch, Dictation, Other search, Farfield, Call-center, YouTube) and one test domain (Telephony), and augmented the training data by randomizing the size of the room, reverberation time, position of the microphone, number of background noise sources (0-4), type of background noise sources (randomly drawn from a large dataset), signal to noise ratio, sampling frequency, and codec. The experimental result was that multi-domain training improves cross-domain generalization. Data augmentation using various noises and codecs harmed the ability of the system to generalize to a noise-free test domain; no experimental result was reported for cross-domain generalization to a noisy test domain.

### 2.2. Learning Strategies for Domain Generalization

ERM minimizes the expected loss, with expectation computed over the joint distribution *P* of the random variables *X* and *Y*; this is a form of optimization sometimes called “stochastic optimization” (Rahimian and Mehrotra, 2019). Stochastic optimization is often contrasted with robust optimization (RO), which minimizes the worst-case error:


(3) $$\begin{array}{l} f_{RO}=\underset{f}{\mathrm{argmin}}\underset{y\in Y\left(x\right)}{\max}L\left(f\left(x\right),y\right), \end{array}$$

where Y(x) is called the *uncertainty set:* it is the set of all possible phonemic transcriptions of the utterance *x*. The problem with RO is that it is excessively conservative. For example, if Y(x) is the set of all phoneme transcripts shorter than some predefined maximum length, and if L measures the string edit distance between *f*(*x*) and *y*, then the solution to Equation (3) is an ASR that generates an empty transcript, regardless of *x*.

Distributionally robust optimization (DRO; Rahimian and Mehrotra, 2019) is a hybrid of stochastic optimization and robust optimization. Rather than minimizing the worst loss over a set of transcripts, DRO minimizes the worst expected loss over a set of distributions. Let e∈E specify an environment, e.g., a language being spoken. The environment specifies a joint distribution between *X* and *Y*, and a corresponding environment-dependent stochastic optimization problem; DRO minimizes the worst-case expected loss over all environments in the environment set E:


(4) $$\begin{array}{l} f_{DRO}=\underset{f}{\mathrm{argmin}}\underset{e\in E}{\max}R^{e}\left(f\left(X\right),Y\right)=\underset{f}{\mathrm{argmin}}\underset{e\in E}{\max}{𝔼}_{e}\left[L\left(f\left(X\right),Y\right)\right], \end{array}$$

### 2.3. Representation Learning for Domain Generalization

This article studies two representation learning strategies: invariant risk minimization and regret minimization. Invariant risk minimization (IRM) seeks explicitly to learn a feature representation that is invariant to changes in the environment. Regret minimization (RGM) assumes that completely invariant risk is impossible, and seeks, instead, to minimize the regret incurred by training on the wrong environments.

#### 2.3.1. Invariant Risk Minimization

DRO may be inefficient if one of the environments is intrinsically more difficult than the others, e.g., if one language is intrinsically more difficult to transcribe. For example, suppose that


$$\begin{array}{l} f_{1}=\underset{f}{\mathrm{argmin}}R^{1}\left(f\left(X\right),Y\right), \end{array}$$

and suppose that $R^{e}\left(f_{1}\right)\leq R^{1}\left(f_{1}\right)$ for all *e* ≠ 1; then the DRO solution is nothing other than the optimal ASR for language 1, and we might as well discard the rest of the training data. Invariant risk minimization (Arjovsky et al., 2019) seeks to find a better balance among the many different languages in the training corpus by computing an invariant embedding *Z* = ϕ(*X*) such that the optimal speech recognizer, *Y* = *w*(*Z*), is the same in all languages.

Invariant risk minimization finds an environment-dependent classifier *f* = *w* ∘ ϕ that is the composition of a feature extractor, ϕ:*X* → *Z*, and a classifier, *w* : *Z* → *Y*. The feature extractor is judged to achieve invariant risk if the minimum-risk classifier sets for all of the environments, $\mathrm{argmin}R^{e}\left(w\right)$, overlap by at least one element: there is at least one classifier that is simultaneously optimal in all environments. Invariant risk minimization finds (*w*, ϕ) that minimize the overall risk, subject to the constraint that ϕ achieves invariant risk:


(5) $$\begin{array}{l} f_{IRM}=\underset{w,\phi }{\mathrm{argmin}}\underset{e\in E}{\sum }R^{e}\left(w○\phi \left(X\right),Y\right), \end{array}$$

$$\begin{array}{ll} \text{s.t. } & w\in \underset{\bar{w}}{\mathrm{argmin}}R^{e}\left(\bar{w}○\phi \left(X\right),Y\right)\;\forall e\in E. \end{array}$$

Equation (5) defines IRM, but is difficult to implement. The constrained optimization in Equation (5) requires that, in order to update the feature extractor, one must determine the update's effect on the set of optimal classifiers in every environment. Arjovsky et al. (2019) propose that finding $w\in \mathrm{argmin}R^{e}$ is equivalent to minimizing the L2-norm of the gradient, $∥\nabla_{w}R^{e}{∥}^{2}$, for every environment, which can be performed using a multi-task learning framework with a weighting coefficient of λ:


(6) $$\begin{array}{l} f_{IRM}=\underset{w,\phi }{\mathrm{argmin}}\underset{e\in E}{\sum }R^{e}\left(w\left(\phi \left(X\right)\right),Y\right)+\lambda ∥\nabla_{w}R^{e}\left(w\left(\phi \left(X\right)\right),Y\right){∥}_{2}^{2}. \end{array}$$

The division of *f* into two subsystems, ϕ and *w*, is somewhat arbitrary; in an end-to-end neural network, any particular layer could be arbitrarily chosen to be trained as the invariant embedding. Arjovsky et al. (2019) take inspiration from the observation that, when the loss function is either mean squared error or cross entropy, the optimal classifier is the conditional expectation of *Y* given ϕ(*X*) (Bishop, 1996). In this case, the feature extractor ϕ is optimal across environments if and only if we have:


(7) $$\begin{array}{l} {𝔼}_{e_{i}}\left[Y|\phi \left(X\right)=z\right]={𝔼}_{e_{j}}\left[Y|\phi \left(X\right)=z\right]\;\forall e_{i},e_{j}\in E, \end{array}$$

Arjovsky et al. (2019) observe that Equation (7) is most simply satisfied if ϕ(*x*) = *z* = *y*. In order to guarantee that ϕ(*x*) = *y* satisfies the condition in Equation (6), they propose fixing *w*(*z*) = *w*·*z*, and fixing the coefficient to *w* = 1.0, thus:


(8) $$\begin{array}{l} f_{IRM}=\;\underset{\phi }{\arg\min}\sum_{e\in ℰ}{ℛ}^{e}(\phi (X),Y)+\lambda \|\nabla_{w:w=1.0}{ℛ}^{e}(w\cdot \phi (X)),Y){\|}_{2}^{2}. \end{array}$$

#### 2.3.2. Regret Minimization

The framework of regret minimization was originally proposed in economics, in order to explain the tendency of human actors to consistently make choices that lead to suboptimal expected rewards (Bell, 1982). The framework of regret minimization proposes that rational actors have reason to doubt their own estimates of the probabilities of future events. One way to compensate for lack of knowledge is by minimizing expected regret, where regret is an increasing convex function of foregone income, such that potential events that lead to a great deal of foregone income are overweighted relative to their estimated probability. Jin et al. (2020) proposed applying regret minimization to the task of domain adaptation in machine learning. They proposed that the distribution of environments in a test corpus is often badly matched to the distribution of environments in a training corpus, and that it is therefore rational to learn a classifier that minimizes the regret incurred by training on the wrong subset of environments.

Denote $R^{e}\left(w○\phi \right)$ as the risk computed over environment *e*, and $R^{-e}\left(w○\phi \right)$ as the risk computed over all environments other than environment *e*, *i.e*.,


(9) $$\begin{array}{l} R^{-e}\left(w○\phi \right)={𝔼}_{e^{\prime }\neq e}\left[L^{e^{\prime }}\left(w○\phi \right)\right] \end{array}$$

Further, define *w*^*e*^ and *w*^−*e*^ as the minimizers of the corresponding risks


(10) $$\begin{array}{l} w^{e}=\underset{h}{\mathrm{argmin}}R^{e}\left(h○\phi \right),\;w^{-e}=\underset{h}{\mathrm{argmin}}R^{-e}\left(h○\phi \right) \end{array}$$

The regret minimization criterion proposed by Jin et al. (2020) is then


(11) $$\begin{array}{l} f_{RGM}=\underset{w,\phi }{\min}R\left(w○\phi \right)+\lambda \underset{e}{\sum }\left[R^{e}\left(w^{-e}○\phi \right)-R^{e}\left(w^{e}○\phi \right)\right] \end{array}$$

The first term in Equation (11) is the empirical risk averaged over all environments. The second term measures the sum, across all environments, of the regret, *R*_*e*_(ϕ), that would be incurred by training and testing on different environments:


(12) $$\begin{array}{l} R_{e}\left(\phi \right)=R^{e}\left(w^{-e}○\phi \right)-R^{e}\left(w^{e}○\phi \right) \end{array}$$

Since *w*^−*e*^ and *w*^*e*^ are minimizers, *R*_*e*_(ϕ) is a function of ϕ. Since *w*^*e*^ is the minimizer of $R^{e}\left(w○\phi \right)$, *R*_*e*_(ϕ) is guaranteed to be non-negative. The minimizer of *R*_*e*_(ϕ), therefore, is a feature extractor that eliminates all information about the environment, in the sense that the cross-environment classifier, *w*^−*e*^, performs exactly as well as the optimum environment-dependent classifier, *w*^*e*^.

IRM (Section 2.3.1) requires that the globally optimum classifier, *w*, must also be a minimizer of the environment-dependent risk for every particular environment: $R^{e}\left(w○\phi \right)=R^{e}\left(w^{e}○\phi \right)$. If the constrained optimization of Equation (5) is solved using a Lagrangian optimization technique, the Lagrangian form is


(13) $$\begin{array}{l} f_{IRM}=\underset{w,\phi }{\min}R\left(w○\phi \right)+\lambda \underset{e}{\sum }\left[R^{e}\left(w○\phi \right)-R^{e}\left(w^{e}○\phi \right)\right] \end{array}$$

The similarities and differences between IRM and RGM may be understood by comparing Equations (11 and 13). Like IRM, regret minimization uses a Lagrangian constraint term to enforce invariance. Unlike IRM, the classifier *w*^−*e*^ is trained without access to samples in environment *e*, so that RGM in theory enforces a stronger invariance constraint on the feature extractor ϕ than IRM: in the terminology of Żelasko et al. (2020), IRM minimizes the difference between multilingual and monolingual error rates, while RGM minimizes the difference between cross-lingual and monolingual error rates.

**Figure 2:**
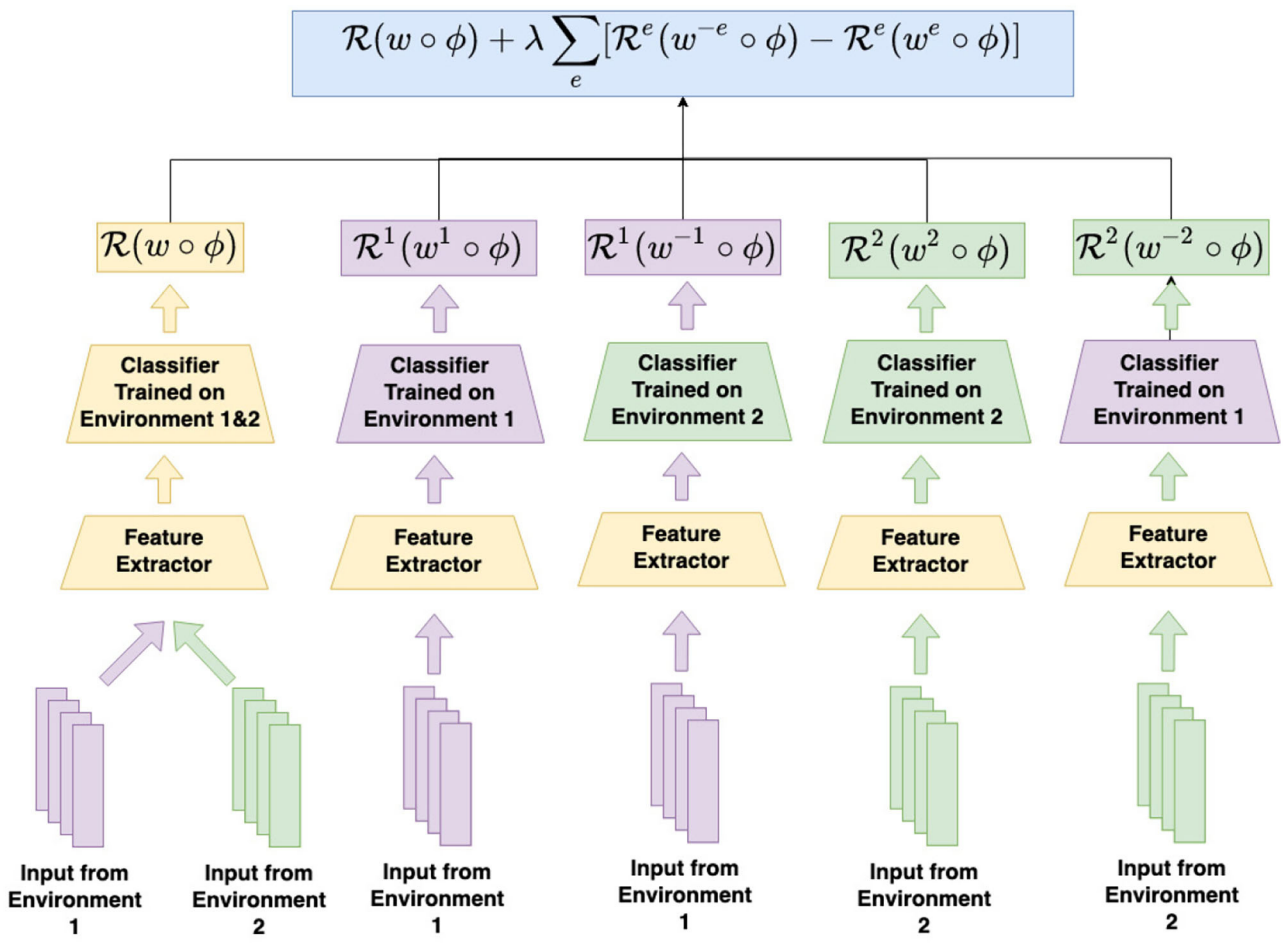
How to compute the risk for regret minimization in a two-environment setting. In addition to the ERM risk calculated on inputs from both environment under the shared feature extractor and classifier, regret minimization inserts an additional regret term for each environment into the total risk. The regret for environment 1, for example, can be calculated by first feeding the inputs from environment 1 into the feature extractor, and then into the classifier trained on environment 1, as well as the classifier trained on all environments *except* environment 1. The difference calculated from the corresponding loss term for the out-of-environment classifier and the within-environment classifier is the regret. The same calculation can be done to calculate the regret for environment 2.

The procedure for regret minimization is schematized in Figure 2. As shown, even with only two distinct training environments (*X*^1^ and *X*^2^), five distinct classifiers must be trained (the globally optimum classifier *w*, the environment-dependent classifiers *w*^1^ and *w*^2^, and the cross-environment classifiers *w*^−1^ and *w*^−2^).

## 3. Algorithms

Language-independent ASR was trained using empirical risk minimization (ERM), distributionally robust optimization (DRO), and invariant risk minimization (IRM) using exactly the algorithms described in Equations (2), (4), and (8), respectively, where e∈E is the language identifier, *x* ~ *X* is a sequence of acoustic feature vectors, and *y* ~ *Y* is a phonemic transcription represented using the symbols of the international phonetic alphabet.

**Figure 3:**
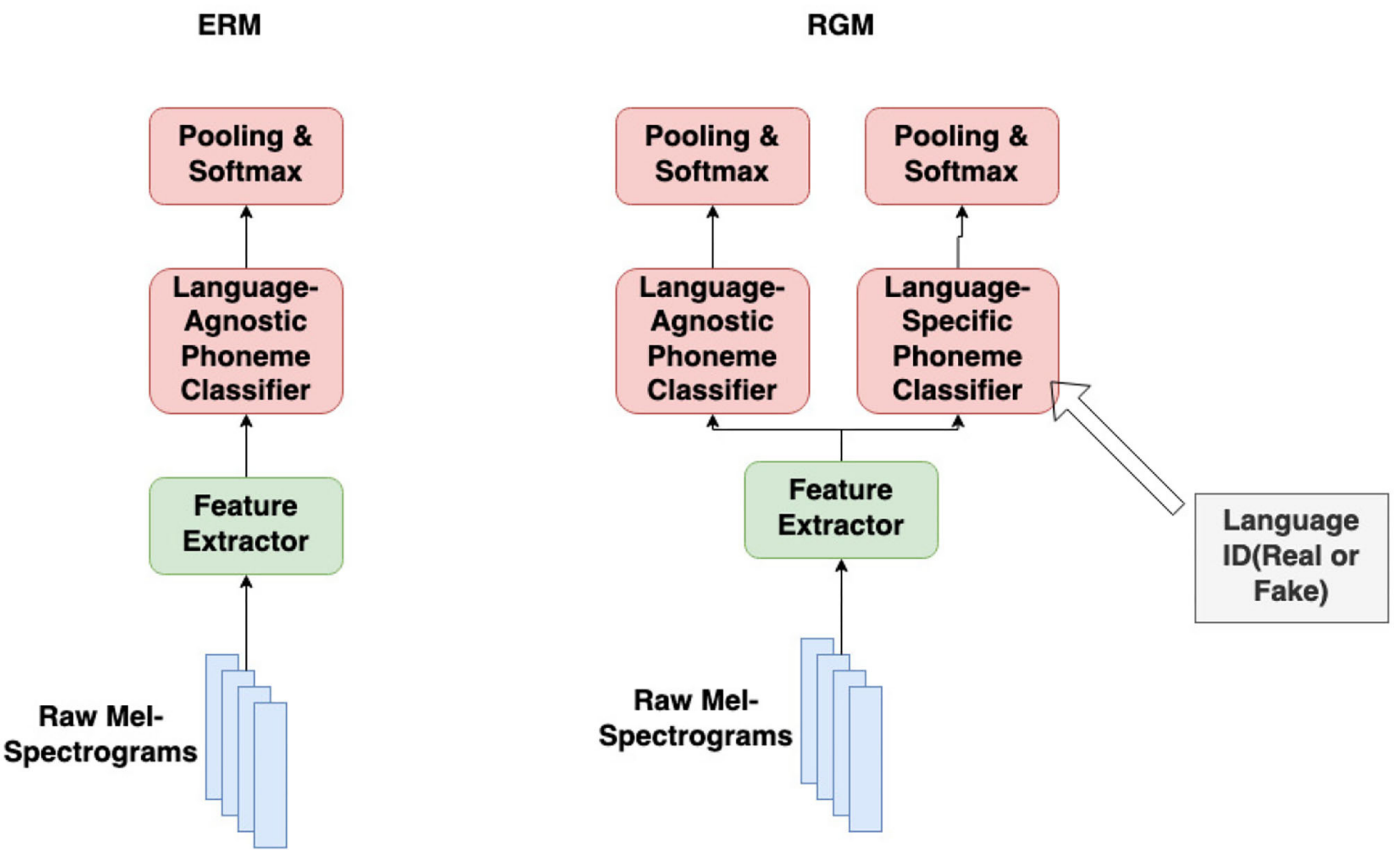
The modified architecture for regret minimization (RGM) vs. the original architecture for empirical risk minimization (ERM). Both methods train a language-agnostic phone token classifier; RGM also trains language-specific phone token classifiers.

The regret minimization method proposed in Equation (11) is computationally impractical for ASR, because it requires optimizing an ASR separately for every leave-one-language-out subcorpus, $w^{-e}={\mathrm{argmin}}_{h}R^{-e}\left(h○\phi \right)$; doing so is impractical when the subcorpus for each training language contains many hours of labeled speech. In order to make regret minimization practical for ASR, we modify Equation (11) into


(14) $$\begin{array}{l} \underset{w,\phi }{\min}R\left(w○\phi \right)+\lambda \underset{e,e^{\prime }:e\neq e^{\prime }}{\sum }\left[R^{e}\left(w^{e^{\prime }}○\phi \right)-R^{e}\left(w^{e}○\phi \right)\right] \end{array}$$

which is essentially replacing the leave-one-out classifier with the single-language classifier on *a different language*. This leaves us with one feature extractor, ϕ(***X***), |E| different single-language phone token classifiers *w*^*e*^(***Z***), and one language-agnostic phone token classifier *w*(***Z***), as shown in Figure 3. Figure 3 compares empirical risk minimization (ERM), which trains only the language-agnostic classifier, to the modified RGM of Equation (14), which also trains language-specific phone token classifiers for each language in the training corpus. Each iteration of training consists of three steps:

1. Feed {***X***^*e*^} into the single-language classifier, and perform *K* steps of gradient descent to find $w^{e}=\mathrm{argmin}R^{e}\left(w○\phi \right)$.
2. Feed {***X***} into the language-agnostic classifier, and perform *K* steps of gradient descent to find w=argminR(w○ϕ).
3. Append a fake language label, *e*′ ≠ *e*, to each utterance. Train ϕ by performing 1 step of gradient descent on
  (15) $$\begin{array}{l} R\left(w○\phi \right)+\lambda \left[R^{e}\left(w^{e^{\prime }}○\phi \right)-R^{e}\left(w^{e}○\phi \right)\right] \end{array}$$

When the classifier *f* = *w* ∘ ϕ is used as a phone token recognizer, its per-frame softmax outputs are scored using connectionist temporal classification (Graves et al., 2006); when used as a phone token classifier, its per-frame logits are mean-pooled and then passed through a softmax nonlinearity, as stated in Figure 3.

## 4. Experimental Methods

### 4.1. Phone Token Recognition

We use ESPnet (Watanabe et al., 2018) as our ASR framework which offers a complete ASR pipeline including data preprocessing, transformer network implementation (Vaswani et al., 2017), network training and decoding. We choose 15 languages as the multilingual set and an additional 5 languages as the cross-lingual set. Models are trained, validated, and tested using languages in the multilingual set; languages in the cross-lingual set are used only for testing. The details of our dataset are listed in Table 1.

**Table 1:** Sources of data used in our cross-lingual experiment.

**Language**	**Abbr**	**Corpus**	**Type**	**Family**	**Len**
Portuguese	por	GP	Read	Romance	26
Turkish	tur	GP	Read	Turkic	17
German	deu	GP	Read	Germanic	18
Bulgarian	bul	GP	Read	South Slavic	21
Thai	tha	GP	Read	Tai	22
Mandarin	cmn	GP	Read	Sinitic	31
French	fra	GP	Read	Romance	25
Czech	ces	GP	Read	West Slavic	29
Dutch	nld	CGN	Read	Germanic	64
Georgian	kat	Babel	Sp.	Kartvelian	190
Javanese	jav	Babel	Sp.	Austronesian	204
Amharic	amh	Babel	Sp.	Ethiopic	204
Zulu	zul	Babel	Sp.	Bantu	211
Vietnamese	vie	Babel	Sp.	Vietic	215
Bengali	ben	Babel	Sp.	Indo-Aryan	215
Croatian	hrv	GP	Read	South Slavic	16
Polish	pol	GP	Read	West Slavic	24
Spanish	spa	GP	Read	Romance	22
Lao	lao	Babel	Sp.	Tai	207
Cantonese	yue	Babel	Sp.	Sinitic	215

*The upper part is the multilingual set and the lower part is the cross-lingual set. “Corpus” is GlobalPhone, corpus of spoken Dutch, or Babel. “Type” column denotes whether the corpus contains spontaneous (Sp.) or read speech. “Len” column shows the total duration of all utterances in hours. “Family” column shows the language family*.

Data are extracted from three publicly available corpora: GlobalPhone (Schultz, 2002), the corpus of spoken Dutch (Schuurman et al., 2003), and Babel (Andrus et al., 2016, 2017; Bills et al., 2016a,b, 2019, 2020; Benowitz et al., 2017; Adams et al., 2019). The former two corpora contain read speech, while Babel contains primarily spontaneous speech.

Due to the sampling rate differences among corpora, we first upsample all audio signals to 16kHz. Using Kaldi, we then extract 80-dimensional log Mel spectral coefficients with 25 ms frame size and 10 ms shift between frames, and augment the frame vectors with 3 extra dimensions for pitch features. The transcriptions are converted to IPA symbols using LanguageNet grapheme-to-phone (G2P) models (Hasegawa-Johnson et al., 2020). Following (Żelasko et al., 2020), ASR is trained end-to-end with an output vocabulary consisting of *phone tokens* instead of phones. A phone token is defined to be any single character in the IPA transcription, including base phones, diacritics, and tone symbols; the Cantonese syllable nucleus [a:ߗ], for example, is decomposed into four phone tokens: /a/, /:/,/˦/, and /⌉/. The resulting phone token inventory contains the 95 distinct IPA characters present in phoneme transcriptions of the 15 training languages. IPA characters present in the test languages, but not in the training languages, are mapped to the out-of-vocabulary (OOV) symbol UNK.

The encoder part of our Transformer network starts with two 2D convolutional layers with a subsampling factor of 4, followed by 12 self-attention encoder layers, each having 4 heads, an attention dimension of 256 and a 2, 048-dim position-wise feed-forward layer. The encoder output is passed through a dense layer to compute frame-wise phone token posteriors, which are scored using connectionist temporal classification (CTC, Graves et al., 2006).

For the experiments involving the Slavic subset, we chose the four Slavic languages (Bulgarian, Czech, and Polish for multilingual training and Croatian for cross-lingual testing) out of the 20-language set. The features used are the same as those from the 20-language experiment, but the label set contains only the phone tokens from the three multilingual training languages. This results in a total of 46 phonetic tokens. For recognition scoring purposes, OOV IPA characters in Croatian are each mapped to the closest token in the phone token inventory. Two additional test languages, French and German, are also used for further evaluation, but any OOV tokens are mapped to UNK instead.

### 4.2. Phone Token Classification

In addition to recognition experiments, we also tested all training algorithms in a phone token classification experiment, using training and test data from only Polish, Bulgarian, Czech, Croatian, French and German. Grapheme-to-phoneme transducers were first applied to the original text transcriptions to obtain IPA transcriptions. IPA transcriptions were then split into individual phone tokens. There are no lexical tones in these six languages, but several of them use other diacritics: the IPA lengthening symbol (/:/) composed 3.2% of all phone tokens, and other diacritics composed 2.3% of the remaining phone tokens (0.3–0.6% each). Triphone hidden Markov models (HMMs) were trained for each language individually, with phone tokens as targets; for example, the triphone /a-:+p/ denotes the sound made by the IPA lengthening symbol (/:/) when it follows the vowel /a/, and precedes the consonant /p/. Kaldi was used to train and cluster the triphones, and to force-align them to audio, in order to find physical segment boundaries for each triphone. Based on the forced alignment, we then segmented variable-length phone token utterances from the audio to construct a multilingual phone token classification dataset. The training set was further subsampled by a factor of 3, leading to 688 k training pairs. We then trained a model consisting of six transformer encoder layers (instead of 12 as in the previous experiments; all other architectural details are the same when applicable) and mean-pooled the time steps to obtain phone token logits, which are fed forward to a single softmax nonlinearity for the entire phone token segment. Phone tokens that appear in the test languages (Croatian, French and German) but not in the training languages (Czech, Bulgarian and Polish) were excluded from the evaluation corpus.

## 5. Results

Table 2 lists phone token error rates (PTER, %) of an ASR trained using 15 languages, and tested on five additional languages. The 15 training languages were chosen to span 10 language families; the five test languages were chosen to be members of five of the same families. Parameters of the ASR were trained using training data in the 15 languages shown in the left column. Each neural network was trained until PTER reached a minimum on development test data in the 15 training languages (a strategy sometimes called *early stopping* Prechelt, 1998). Other hyperparameters, including multi-task training weights for IRM and RGM, were also optimized for minimum error on development test data in the training languages. The results reported in Table 2 were then measured using evaluation test data in both training and test languages. As shown, ASR trained using empirical risk minimization (ERM, Equation 2) gave the best results for every training language, with a large relative advantage. For languages that were not part of the training set, ERM is still better than other training methods, but its advantage is much smaller.

**Table 2:** Phone token error rates (PTER, %) of an ASR trained on 15 languages, tested on 5 additional languages.

**Training languages**					**Test languages**				
**Language**	**ERM**	**DRO**	**IRM**	**RGM**	**Language**	**ERM**	**DRO**	**IRM**	**RGM**
Portuguese	**18.4**	22.6	20.5	22.1	Croatian	**47.8**	48.9	49.3	50.9
Turkish	**21.3**	23.0	24.0	25.0	Polish	62.5	**62.2**	63.7	65.5
German	**26.1**	28.4	27.2	29.4	Spanish	**38.1**	39.8	39.6	40.6
Bulgarian	**27.0**	30.0	30.1	30.2	Lao	**78.2**	**78.2**	79.0	78.8
Thai	**26.1**	30.0	31.3	34.5	Cantonese	**77.0**	78.0	78.4	77.7
Mandarin	**30.0**	38.5	33.8	46.3	-	-	-	-	-
French	**13.7**	19.1	16.3	16.8	-	-	-	-	-
Czech	**11.0**	15.6	12.8	13.7	-	-	-	-	-
Dutch	**21.3**	28.7	28.3	27.6	-	-	-	-	-
Georgian	**38.0**	43.9	46.6	41.5	-	-	-	-	-
Javanese	**47.0**	54.4	55.6	49.6	-	-	-	-	-
Amharic	**44.7**	52.2	53.0	49.7	-	-	-	-	-
Zulu	**42.4**	48.9	48.9	46.3	-	-	-	-	-
Vietnamese	**52.3**	59.1	63.1	58.5	-	-	-	-	-
Bengali	**40.2**	47.0	47.4	43.4	-	-	-	-	-
**Average**	**30.6**	36.1	35.9	35.6	**Average**	**60.7**	61.4	62.0	62.7

*Early-stopping epoch and other hyperparameters of each algorithm were selected based on development test data in the training languages. Numbers reported are from the evaluation test data in each language. Bold denotes lowest error in each row*.

Table 3 lists PTER (%) of an ASR trained using three languages from the Slavic language families. The ASR was also tested on one Slavic test language (Croatian), and two non-slavic Indo-European languages (French and German). Early stopping and hyperparameter optimization were performed using development test data in the training languages. Table 3 reports PTER measured using evaluation test data in all six languages. The results are quite different from those shown in Table 2. ERM achieves the lowest error rates on the three training languages, and on the test language that is drawn from the same language family (Croatian), but both French and German achieve lower error rates using regret minimization.

**Table 3:** Phone token error rates (PTER, %) of an ASR trained on three Slavic languages (Czech, Bulgarian and Polish).

	**Training languages**				**Test languages**			
**Algorithm**	**Czech**	**Bulgarian**	**Polish**	**Average**	**Croatian**	**French**	**German**	**Average**
ERM	**26.4**	**41.7**	**44.9**	**37.7**	**56.4**	71.5	65.4	64.4
DRO	37.2	49.7	51.1	46.0	60.6	75.0	67.8	67.8
IRM	34.0	47.3	50.2	43.8	57.7	70.3	66.7	64.9
RGM	32.3	46.0	48.2	42.2	57.1	**69.2**	**65.3**	**63.9**

*Early-stopping and other hyperparameters of each algorithm were selected based on development test data in the three training languages. Numbers reported are from the evaluation test data in each of the three training languages, and in each of three previously unseen test languages. Bold denotes lowest error in each column*.

Table 4 lists phone token classification error rates (PTCER) for the same six languages listed in Table 3. As described in Section 4.2, these experiments were performed by segmenting each audio file using forced alignment with a monolingual phone-token HMM ASR. The resulting phone token segments were then classified using a Transformer-based phone token classifier, whose parameters, hyperparameters, and early-stopping schedule were optimized using training data and development test data from Bulgarian, Polish, and Czech. Results are shown for a range of values of the IRM multi-task learning weight, λ (see Equation 8) for precise definition of this hyperparameter). It is shown that the optimal value of λ calculated using the training languages (λ = 10) is also optimal for the test language that is a member of the same language family (Croatian), and is optimal on average across all three test languages, but is not optimal for either French or German individually.

**Table 4:** Phone token classification error rates (PTCER, %) of an ASR trained on three Slavic languages (Czech, Bulgarian and Polish).

	**Training languages**				**Test languages**			
**Algorithm**	**Czech**	**Bulgarian**	**Polish**	**Average**	**Croatian**	**French**	**German**	**Average**
ERM	**29.7**	46.2	42.9	39.6	48.3	56.6	59.3	54.7
DRO	40.7	51.5	46.1	46.1	50.7	**55.6**	60.1	55.5
IRM, λ = 0.001	34.6	49.9	43.5	42.7	49.0	57.6	59.8	55.5
IRM, λ = 0.01	34.7	49.6	43.3	42.5	48.2	57.4	59.3	55.0
IRM, λ = 0.1	34.8	49.8	43.3	42.6	48.2	57.3	59.1	54.9
IRM, λ = 1	35.6	50.8	43.5	43.3	49.1	57.1	60.1	55.4
IRM, λ = 10	30.7	**45.6**	**41.3**	**39.2**	**46.2**	55.8	59.1	**53.7**
IRM, λ = 100	41.1	51.5	48.6	47.1	47.6	55.9	**58.7**	54.1
RGM	32.0	49.0	45.7	42.2	48.8	57.3	64.3	56.8

*Early-stopping and other hyperparameters of each algorithm were selected based on development test data in the three training languages. Numbers reported are from the evaluation test data in each of the three training languages, and in each of three previously unseen test languages. Bold denotes lowest error in each column*.

Table 5 lists phone token classification error rates (PTCER) for Transformer-based phone classifiers trained exactly as in Table 4, except that training is stopped in a different manner. In Table 4, training was stopped when PTCER reached a minimum on development test data in the training languages. In Table 5, however, training was stopped when PTCER reached a minimum on development test data in one of the test languages. Numbers in boldface in Table 5 highlight the best results achieved when parameters are trained in (three) training languages, early-stopping is timed using a (fourth) development-test language, and then the system is evaluated in a (fifth) evaluation-test language. As shown, early-stopping using a development-test language outperforms early-stopping using a training language in two of the three languages.

**Table 5:** Phone token classification error rates (PTCER, %) of an ASR trained on three Slavic languages (Czech, Bulgarian and Polish) and tested on one Slavic language (Croatian) and two other Indo-European languages (French and German).

	**Early-stopping**	**Eval languages**		
**Algorithm**	**language**	**Croatian**	**French**	**German**
ERM	Croatian	46.6	59.4	63.4
RGM	Croatian	44.9	**56.2**	**57.2**
ERM	French	46.8	58.4	60.6
RGM	French	47.5	56.0	60.5
ERM	German	48.9	62.2	59.3
RGM	German	**44.9**	**56.2**	57.2

*In this table, the epoch for early stopping was chosen using development-test data from one of the three test languages: Croatian in rows 1–2, French in rows 3–4, German in rows 5–6. PTCER was then measured using evaluation-test data from each test language. Numbers reported using early-stopping on the test language are considered oracle; boldface shows the lowest non-oracle error rate*.

## 6. Discussion

Empirical risk minimization (ERM) is provably optimal, in the limit of infinite training data, if the test data are drawn from the same distribution as the training data, e.g., when training and test data are drawn from the same set of languages. DRO, IRM, and RGM each seek to compensate, during training, for possible differences between the training languages and the test languages. DRO seeks to enforce generalizability by minimizing the maximum error rate, where maximization is performed across all training languages. IRM seeks to enforce generalizability by forcing the ASR to find a solution that is simultaneously optimal in all training languages; in order to find a solution that is optimal in all training languages, the ASR may be forced to discard information that would make the optimal classifier different in one language or another. RGM seeks to enforce generalizability by minimizing the differences between crosslingual and monolingual error rates (termed the “regret”).

Three hypotheses were proposed in Section 1; this section discusses the status and interpretation of those hypotheses, in light of the experimental results in Section 5.

- **H1:** Domain-invariant machine learning methods such as DRO, IRM, and/or RGM can be used to optimize E2E ASR so that it generalizes from 15 training languages to five novel test languages more effectively than if it were trained using ERM. **Status:** False.

Hypothesis **H1** is falsified by the experimental results in Table 2. The conclusion suggested by this result is that the training data and the test data are drawn from the same distributions. For example, we might (speculatively) conclude that the distribution of speech sounds in these five test languages is reasonably well represented by the set of 15 training languages.

- **H2:** DRO, IRM, and/or RGM, as compared to ERM, can be applied to optimize an E2E ASR so that it more effectively generalizes from training languages in one language family to test languages in a different language family. **Status:** True.

Experimental results in Table 3 suggest that hypothesis **H2** is true. In the experiment described in Table 3, regret minimization (RGM) is used to minimize the difference between crosslingual and monolingual error rates of languages in the same family (Slavic). The resulting trained parameters can be applied to languages from other language families (French and German) with better results than the results achieved using ERM.

- **H3:** The optimal training regimen for phone token classification (given known phone token boundary times) is different from the optimal training regimen for phone token recognition (with unknown boundary times). **Status:** True.

Experimental results in Tables 4, 5 suggest that hypothesis **H3** is true. The recognition error rates shown in Table 3 are optimized by ERM (if the test language is in the same family as the training language) or RGM (otherwise). The classification error rates in Table 4, on the other hand, are optimized using IRM. IRM forces the recognizer to discard some of the information from the input, so that the scale of the output softmax layer is simultaneously optimal in every training language. It is possible that forcing language-invariance of the output layer, as performed by IRM [Equation (8)], is effective when there is a single output layer computing classification results for the entire segment, but is ineffective in an ASR system in which the output layers of multiple frames are combined using CTC (Graves et al., 2006). This conclusion is supported by the results in Table 5, in which the early-stopping schedule was governed by a test language rather than by the training languages. Test-based early-stopping improved the performance of RGM, relative to Table 4, and was able to outperform the best IRM results in two of the three test languages.

## 7. Conclusions

Empirical risk minimization (ERM) is asymptotically optimal when the training data and test data are drawn from the same distribution, e.g., when training and test data are drawn from the same languages. When training and test data are drawn from different languages, the optimal training regimen depends on the number of training languages, the existence or absence of familial relationships between training and test languages, and the type of recognition algorithm.

An ASR trained using 15 training languages from 10 language families, and tested using other languages from the same families, can be effectively trained using ERM. Apparently, in this situation, the distribution of speech sounds in the test languages is reasonably well represented by the distribution of speech sounds in the training languages.

An ASR trained using three languages from one language family, and tested using a fourth language from the same family, can be effectively trained using ERM. When the test languages are drawn from other language families, however, the generalization ability of the recognizer can be enhanced by a method called regret minimization. Regret minimization trains the recognizer to minimize the difference between crosslingual and monolingual error rates.

Cross-lingual phone token classification is optimized using a method called invariant risk minimization. IRM forces the classifier to generate an output softmax layer whose scale is simultaneously optimal in all training languages. Speculatively, it is possible that IRM is optimal for phone token classification, but not for phone token recognition, because the softmax-normalization step in IRM is poorly matched to the CTC training criterion used in ASR.

## Data Availability Statement

Publicly available datasets were analyzed in this study. This data can be found at: https://www.ldc.upenn.edu/; http://www.elra.info/; http://lands.let.ru.nl/cgn.

## Author Contributions

The application of IRM to ASR was derived by SC, YZ, and HG. The application of RGM to ASR was derived by YZ, KQ, and JN. The application of DRO to ASR was derived by MH-J, YZ, and HG. HG performed all experiments using the DRO and IRM training criteria and wrote first drafts of Sections 2.2, 2.3.1, and 4.1. JN performed all experiments using the RGM training criterion and wrote first drafts of Sections 2.3.2 and 4.2. MH-J wrote first drafts of Sections 1, 2.1, 5, and 6. All authors contributed to revision of all sections.

## Funding

Work described in this article was funded by a grant from the IBM-Illinois Center for Cognitive Computing Systems Research (C3SR).

## Author Disclaimer

All results and opinions are those of the authors, and are not endorsed by C3SR.

## Conflict of Interest

The authors declare that the research was conducted in the absence of any commercial or financial relationships that could be construed as a potential conflict of interest.

## Publisher's Note

All claims expressed in this article are solely those of the authors and do not necessarily represent those of their affiliated organizations, or those of the publisher, the editors and the reviewers. Any product that may be evaluated in this article, or claim that may be made by its manufacturer, is not guaranteed or endorsed by the publisher.

## Footnotes

^1^The notation *x*_*i*_ ~ *X* means that *x*_*i*_ is an instance of the random variable *X*.
